# Fully automated system for the quantification of human osteoarthritic knee joint effusion volume using magnetic resonance imaging

**DOI:** 10.1186/ar3133

**Published:** 2010-09-16

**Authors:** Wei Li, François Abram, Jean-Pierre Pelletier, Jean-Pierre Raynauld, Marc Dorais, Marc-André d'Anjou, Johanne Martel-Pelletier

**Affiliations:** 1 ArthroVision Inc., 1871 Sherbrooke Street East, Montreal, Quebec H2K 1B6, Canada; 2 Osteoarthritis Research Unit, University of Montreal Hospital Research Centre (CRCHUM), Notre-Dame Hospital, 1560 Sherbrooke St. East, Montreal, Quebec H2L 4M1, Canada; 3 StatSciences Inc., 60 rue Sylvio Leduc, Notre-Dame de l'Île Perrot, Quebec J7V 7P2, Canada; 4 Department of Clinical Sciences, Faculty of Veterinary Medicine, University of Montreal, 3200 Sicotte, Saint-Hyacinthe, Quebec J2 S 2M2, Canada

## Abstract

**Introduction:**

Joint effusion is frequently associated with osteoarthritis (OA) flare-up and is an important marker of therapeutic response. This study aimed at developing and validating a fully automated system based on magnetic resonance imaging (MRI) for the quantification of joint effusion volume in knee OA patients.

**Methods:**

MRI examinations consisted of two axial sequences: a T2-weighted true fast imaging with steady-state precession and a T1-weighted gradient echo. An automated joint effusion volume quantification system using MRI was developed and validated (a) with calibrated phantoms (cylinder and sphere) and effusion from knee OA patients; (b) with assessment by manual quantification; and (c) by direct aspiration. Twenty-five knee OA patients with joint effusion were included in the study.

**Results:**

The automated joint effusion volume quantification was developed as a four stage sequencing process: bone segmentation, filtering of unrelated structures, segmentation of joint effusion, and subvoxel volume calculation. Validation experiments revealed excellent coefficients of variation with the calibrated cylinder (1.4%) and sphere (0.8%) phantoms. Comparison of the OA knee joint effusion volume assessed by the developed automated system and by manual quantification was also excellent (r = 0.98; *P *< 0.0001), as was the comparison with direct aspiration (r = 0.88; *P *= 0.0008).

**Conclusions:**

The newly developed fully automated MRI-based system provided precise quantification of OA knee joint effusion volume with excellent correlation with data from phantoms, a manual system, and joint aspiration. Such an automated system will be instrumental in improving the reproducibility/reliability of the evaluation of this marker in clinical application.

## Introduction

Joint effusion is frequently associated with articular disorders. In osteoarthritis (OA), the effusion is an important marker of the disease flare-up, and its quantification could be helpful as a treatment outcome measure. The most common means used to quantify joint effusion volume is arthrocentesis. A major drawback of this method, however, in addition to being invasive and somewhat painful, is that it often fails to estimate the total joint effusion volume accurately [1]. Several methods for the calculation of the "true" joint effusion volume have been described, and these rely on urea concentration [2], dilution [3,4], or the constant rate of elimination effusion of an injected radioactive tracer by using a radiolabeled albumin technique [5]. However, none of these methods has emerged as a reliable standard.

In the past decade, important progress has been made in the development of magnetic resonance imaging (MRI) technology. Methods have been developed to assess quantitatively or semiquantitatively the structural changes that occur in the joint tissues, including the cartilage, synovial membrane, subchondral bone, and menisci, during OA [6-12]. Other methods have also been reported to allow the estimation of the synovial effusion volume of the knee [13-16] and of the hip [17]. However, these methods are based on manual identification of synovial effusion, and some of them use contrast agents such as gadolinium. In the context of clinical or epidemiologic studies including a large number of patients and in which MRI examinations are done repeatedly over time, or both, the use of contrast enhancement is to be discouraged, as it is invasive and can induce severe complications.

To date, the methods reported using MRI to assess joint effusion have used manual segmentation. To the authors' knowledge, no other report exists on any automated technology available for such a purpose. Herein, we describe the methodology of a fully automated system performed on MR images, without the use of a contrast agent, to assess quantitatively the joint effusion volume in human OA knees. In addition, three validation protocols of the technology were performed comparing the volumes obtained by using the developed automated system.

## Materials and methods

### MRI protocol

MRI was conducted on a 1.5-T whole-body scanner (Magnetom Avanto, Siemens, Erlangen, Germany) by using a knee coil. The MRI examination consisted of the two sequences in axial planes without patient repositioning: T2-weighted gradient-echo true-fast-imaging-with-steady-state-precession sequence (T2-trueFISP, TR/TE: 6/3 ms, ST/SS, 3/0 mm; FOV, 160 mm; FA, 90 degrees; NEX, 2; matrix, 320 × 320 px; reconstructed image, 320 × 320 px; voxel size, 0.5 × 0.5 × 3 mm^3^); and T1-weighted inphase-outphase gradient-echo (GRE) sequence (in-TR/TE, 450/2.6 milliseconds; out-TR/TE, 450 milliseconds/6.4 milliseconds; ST/SS, 3/0 mm; FOV, 180 × 144 mm; FA, 70 degrees; NEX, 1; matrix, 320 × 256 px; reconstructed image, 640 × 512 px; and voxel size, 0.28 × 0.28 × 3 mm^3^); herein referred to as T2 and T1 sequences, respectively. The acquisition time of each of these sequences was approximately 7 minutes and consisted of about 35 slices.

### Patient description

Twenty-five OA patients (10 M/15 F) aged from 50 to 80 years (68.6 ± 8.4 years; mean ± SD) enrolled in a clinical study were used. OA patients were recruited according to the American College of Rheumatology clinical criteria [18]. Only patients having a Kellgren Lawrence [19] grade ≥ 1 and clinical evidence of joint effusion were included in the study. The study was approved by an institutional medical ethics review board (Institutional Review Board Services (IRB), Toronto, Ontario, Canada). A written informed consent was obtained from each patient. Knees were scanned by using the above described MRI protocol, and a quality-control process was performed on each MR image.

### Joint aspiration

Joint aspiration was performed by a certified rheumatologist and done via the lateral suprapatellar approach. During arthrocentesis, as much joint fluid as possible was aspirated, and the volume was recorded. The arthrocentesis was performed on the same day as, or the day after, the MRI.

### MRI manual joint effusion volume quantification

Joint effusion volume was assessed by using a manual selection in T2 MRI. A reader with more than 4 years of experience in MR image analysis selected the voxels of the joint effusion sites on the images in the T2 sequence by using a voxel selection tool. The joint effusion volumes were then computed and calculated by multiplying the number of selected voxels by the image voxel size, as described by Heuck *et al. *[13]. The intrareader correlation coefficient was excellent (*r *= 0.935; *P *< 0.0001).

### MRI automated joint effusion volume quantification

The joint effusion is described as a 3D object of interest whose discrimination in the MR images is referred to as the "segmentation process." This process was based on 3D processing to benefit from a global and stable approach, although a 2D approach was used for local analysis in some processing to enhance accuracy.

#### Automated bone segmentation

In the context of knee joint effusion segmentation, bones (femur and tibia) were first segmented in the T1 sequence and used as a stable reference. Thresholding, a basic image segmentation method, was used to select the voxels of the bones. Because the intensity distribution of the bones stood in the mid-range of the intensities of the image, two thresholds were needed, a lower and an upper threshold, to define the bone intensity interval. In brief, in knee MRI, the 3D acquisition was adjusted around the joint; thus the femur was located consistently at the center of the images for all patients. This enabled defining, in the same image position, a small region (18 × 36 × 18 mm^3^) inside the femur. The two thresholds were automatically computed by the analysis of the intensity histogram of this defined 3D region, with a widely used technique to search for an optimal point on an intensity histogram curve [20] (Support material 1 in Additional file 1). A 2D technique of intraimage contrast analysis was further used (Support material 2 in Additional file 1) to correct the bone boundaries on the image signal. Finally, an interimage repairing process was used, allowing adjustments where object information was missing or partial (Support material 3 in Additional file 1). The T1 segmented bones were then transported into the T2 sequence local coordinate to make these results available for the T2 processing stages. This was done by using the pixel-to-3D transformation matrix stored in each sequence and led to a solid 3D bone object.

#### Filtering of objects of noninterest

The following stage performed in the T2 images consisted of prefiltering large and bright objects other than joint fluid to reduce detection noise. This stage enabled us to mask the objects of noninterest (that is, femur, tibia, patella, and fat), and generated a new T2 image set called "masked images set" used to segment the joint fluid. In this stage, patella and fat tissue were segmented and filtered out together, as they were located in very close proximity. The segmentations were performed in each T2 image by thresholding based on the intensity histogram analysis (Support material 1 in Additional file 1). Thus, the set of obtained segmented images represented the masked images set.

#### Segmentation of joint effusion

The segmentation of joint effusion was performed in the masked image set. Once again, the threshold based on the intensity histogram technique was used for an initial joint effusion segmentation. However, the processing in this case was recursive (that is, a first evaluation allowed delimiting an intensity range of the histogram for which a second evaluation of the optimal point led to the threshold). This method can, however, lead to a possible oversegmentation of the fluid. In other words, in addition to the joint fluid, blood and hyaline cartilage might be selected. Thus, to discard the objects of noninterest, we implemented anatomic filtering by using the relative position of the selected 3D objects to the bones. To complete the segmentation, the interimage repairing technique (Support material 3 in Additional file 1) was also implemented to improve the precision of the detected joint effusion boundaries.

#### Joint effusion volume calculation

The volume calculation used a surface model followed by a subvoxel volume evaluation of the model. This model consists of a triangular mesh composed of triangular facets linking the centers of selected peripheral voxels of the object. This creates a smooth surface in which the volume was calculated with subvoxel precision by counting all the voxels completely inside the object and multiplying the sum by the voxel volume, and for the voxels intersected with the model, only the part of the voxel inside the model was considered, and its subvoxel volume was computed. The object volume was then determined as the sum of all the whole and partial voxel volumes.

### Phantoms

Two types of calibrated phantoms were used to validate the developed automated system. First, a cylinder phantom composed of two cylinders perpendicular to each other filled with a solution of nickel sulfate hexahydrate (NiSO_4_•6H_2_O, 1.25 g/L) was embedded in a container filled with water. The two cylinders were calibrated to a total volume of 14.1 ml. The cylinders were imaged in a 1.5-T whole-body scanner by using a knee coil, according to the described T2 sequence acquisition protocol. Acquisitions were carried out on two different types of apparatus (Signa Excite, GE, Milwaukee, WI, USA, and Magnetom Symphony, Siemens, Erlangen, Germany) and at four different MRI sites. Each site imaged the phantom 50 times over a one-year period. Second, sphere phantoms consisting of a calibrated solid sphere surrounded by the previously mentioned solution (total volume, 247 ml) were imaged five times by using a Siemens apparatus (Magnetom Symphony) at one site, over a period of one year. Quality control was performed for each phantom MR image to ensure the entire presence of the phantom in the image.

### Statistical analysis

Phantom liquid volumes from the MR images and patients' joint effusion volumes from MR images and from direct aspiration were expressed as the mean ± SD. For joint effusion volume, Pearson's correlation was used for the comparison between automated and manual quantifications, and between automated quantification and arthrocentesis. Linear regression analysis also was carried out for the comparison between the joint effusion volume assessed by the automated system and by direct aspiration.

## Results

### MRI sequence acquisition

**Figure 1:**
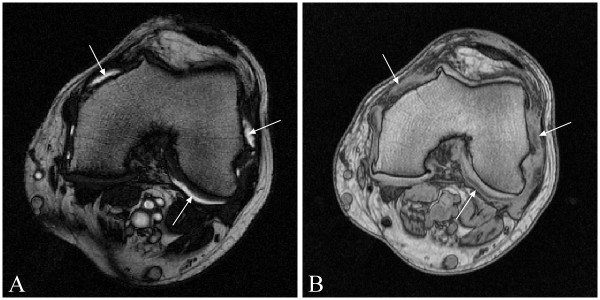
**Representative axial image slices intersecting the femoral condyle**. **(A) **T2 image enhancing the synovial fluid (arrows). **(B) **T1-image showing bright intensity of the femur, in which the synovial membrane (arrows) contrasts against other surrounding tissue.

The MRI examination protocol included T2- and T1-weighted axial sequences (Figure 1). The two MRI sequences used in this study were initially designed for synovial membrane thickness assessment [11]; the T1 sequence enhances the intensity of adjacent tissues such as bone marrow, fat, and synovial membrane, and the T2 sequence enhances the intensity of the joint effusion (Figure 1). These sequences were designed and optimized for visual qualification of the tissues. Among the sequences that were available to enhance the liquid brightness were the PD-TSE, T2-TSE, and T2-trueFISP. Preliminary evaluations were done to compare the images from these three sequences. Data showed that the T2-TSE sequence was associated with truncation artefacts within the joint effusion, whereas the PD-TSE did not provide adequate intertissue contrast, thus making segmentation very difficult. Conversely, tissue contrast was optimal on T2-trueFISP images, enhancing the presence of liquid, while avoiding the presence of truncation artefacts. However, to ensure the acquisition of the entire synovial pouch, the slice number was adjusted on the pouch size evaluated from T2 scout sequences rather than on bone anatomic landmarks.

### Knee effusion volume quantified in MR images with the automated system

The automated joint effusion volume quantification was developed as a four-stage approach to address two challenges; the intensity inhomogeneity artefact (IIH) and the variable topology of the synovial effusion object.

**Figure 2:**
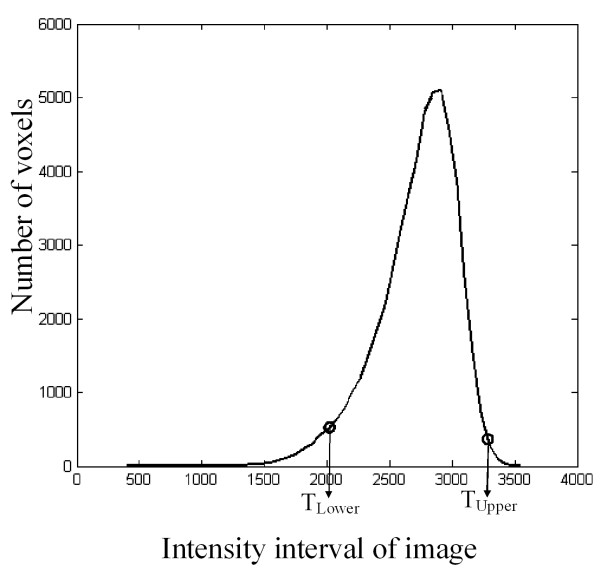
**Illustration of the intensity histogram location of the two thresholds *T_Lower _*and *T_Upper _*of the small 3D region predefined in the femur**. These thresholds were computed as described in Appendix 1 and used to select the bone in the 3D T1-image.

Both femur and tibia were first processed, as the synovial pouch extends anteroproximally on one third of the femur and posterodistally on one fourth of the tibia from the knee. The bone segmentation was performed in the T1 sequence images, in which the bone marrow voxels were more homogeneous. The histogram of the small 3D region predefined in the femur allowed the evaluation of the two thresholds (Figure 2) for each MR image. The 2D contrast of intraimage analysis with the combination of a 2D intensity based interimage repairing technique allowed precise delineation of the bone marrow-to-subchondral bone interface. Once segmented, the bone objects were transported into the T2 image coordinate system.

**Figure 3:**
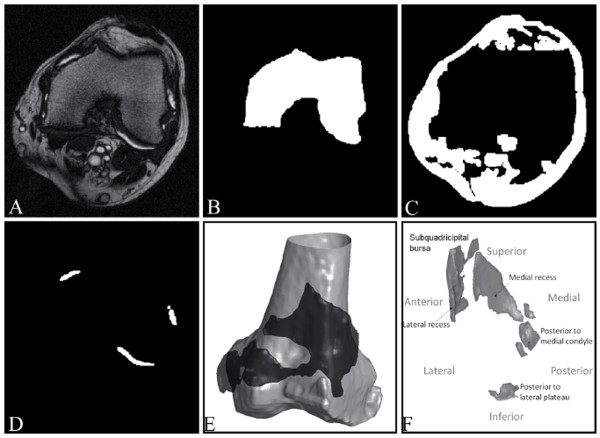
**From MR image to synovial fluid 3D object, illustrations of the results of the main steps of synovial fluid segmentation**. **(A) **Representative knee osteoarthritis patient MRI slices acquired from T2 sequence. Representation of the segmented intermediate results in binary images (white): **(B) **femur, **(C) **other nonfluid objects, and **(D) **joint effusion. **(E, F) **Representations of joint effusion (black in (E)) from two patients and in (E) surrounding the 3D femur (grey).

The second stage consisted of the anatomic filtering of unrelated structures in the T2 images (Figure 3A) by using the segmented bones (Figure 3B) as reference. This stage was completed by the identification of the patella and the fat tissues (Figure 3C) to produce a masked image set that reduced the region of interest for the fluid segmentation.

In the third stage, the threshold computed in the intensity histogram of the masked image set allowed the segmentation of the synovial effusion. A last repairing step helped to prevent the effects of IIH on each of the final objects, allowing the precise detection of the objects' boundaries (Figure 3D). Figures 3E and 3F show 3D representations of the synovial effusion.

**Figure 4:**
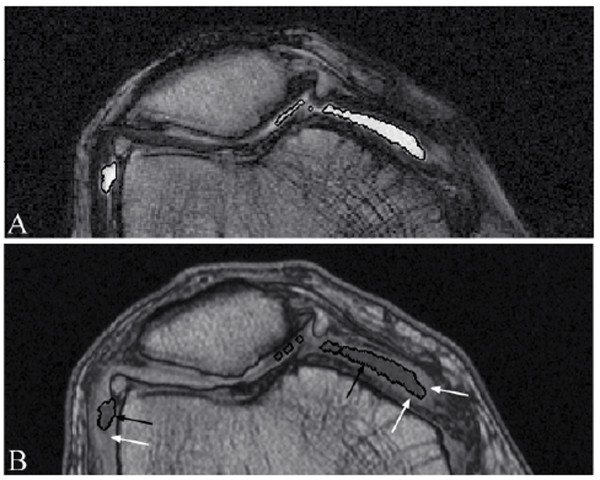
**Synovial fluid 3D object presented in both T1 and T2 MR images**. **(A) **Synovial effusion segmentation (white contours) of a representative knee osteoarthritis patient MRI T2 image slice and **(B) **transported into the corresponding T1 image (black contours), showing that the synovial membrane (bright tissue; white arrows) is excluded from the joint effusion objects (black arrows).

To demonstrate that the segmented joint effusion does not include the synovial membrane, complementary processing was performed in which the joint effusion object was transported from the T2 local coordinate (Figure 4A) to the T1 local coordinate (Figure 4B). Data showed that the synovial membrane was excluded from the joint effusion.

The fourth stage consists of the synovial effusion volume calculation. The average automated computation time to determine the synovial effusion volume fully is about 45 minutes.

### Validation of the automated joint effusion volume quantification

#### Theoretic and automated phantom volume quantification

The cylinder and the sphere phantom images were processed by the developed automated volume quantification system. As no bone or structure is present other than the contour of the phantoms, and these are well contrasted, the process begins at the third stage of the procedure.

For the cylinder phantom (200 acquisitions; 50 independent scans in four MRI centers), data revealed a mean volume of 14.0 ± 0.2 ml compared with the theoretic volume of 14.1 ml, with a coefficient of variation of 1.4%. No true difference was found between each site, and values of 14.1 ± 0.1 ml, 14.1 ± 0.1 ml, 14.0 ± 0.2 ml, and 13.9 ± 0.1 ml were recorded. Similar observations were made when a larger volume was measured. Hence, the sphere phantom (five independent scans at one site) also showed a very low coefficient of variation, 0.8%, with a mean of 246.6 ± 2.0 ml compared with the added theoretic volume of 247 ml.

#### Comparison of MRI OA knee effusion volume assessed by the automated system and by the manual system

The automated quantification of joint effusion from all 25 OA patients gave a mean of 11.7 ± 7.8 ml, whereas 13.5 ± 8.8 ml was obtained by using the manual quantification. The mean absolute difference between automated and manual was 1.8 ± 1.8 ml with excellent correlation coefficient (*r *= 0.98; *P *< 0.0001).

#### Comparison of MRI OA knee effusion volume assessed by the automated system and by direct aspiration

Direct aspiration of the joint effusion in the knee was performed on a subgroup of ten OA patients. A mean value of 12.6 ± 9.8 ml was recorded when the effusion volume was assessed with the automated system compared with 4.1 ± 3.3 ml with the direct aspiration. Correlation between these two data sets was excellent (*r *= 0.88; *P *= 0.0008). Linear regression using the direct aspiration as predictor revealed that the joint effusion volume was 3.4 times greater when determined by MRI compared with direct aspiration.

## Discussion

We report the development and validation of an automated system for quantitative volume determination of knee joint effusion in MR images. Two protocols designed to validate the developed technology, one using calibrated phantoms and another aiming at comparison with a manual technique, showed excellent reproducible results. Further, comparison between the joint effusion volume obtained by MRI followed by quantification with the developed automated system and the direct aspiration performed on knee OA patients also demonstrated an excellent correlation.

The MRI protocol included a T1- and a T2-weighted axial sequence. The choice of axial sequences resulted from the necessity to reduce partial volume effect on the segmentation of the joint effusion. Because of the main orientation of the synovial pouch along the bones, the other acquisition planes, sagittal and coronal, would have produced more partial volume.

Although the T2 sequence enhanced the joint effusion signal, the other fluid-like tissues, such as blood, some hyaline cartilage, and bone marrow lesions, also appeared bright. Consequently, the use of intensity property only would not be sufficient to segment the joint effusion reliably. Thus, the bone was used as an anatomic reference for 3D object filtering. However, as the T2 sequence does not provide a maximal contrasted signal for the bone, the T1 sequence was used for the segmentation of the femur and tibia, as they appear clearer and more homogeneous on the image.

In MR images, inter- and intraimage individual tissue intensities may vary, sometimes greatly. This artefact, known as intensity inhomogeneity (IIH) of a tissue, is caused mostly by the variable amount and location of fluid in each image. To challenge this artefact and to overcome the variable topology of the synovial effusion object, the developed automated system consists of four major stages: (1) segmentation of the bones, (2) filtering of objects of noninterest, (3) joint effusion segmentation, and (4) volume calculation.

The bones (femur and tibia) were first segmented because they play a central role as stable elements and provide excellent anatomic reference for further joint tissue identification. Hence, for the bone segmentation, we used a dynamic threshold calculation, which allowed a threshold evaluation specific to each image quality, providing optimal results for each MRI examination.

Further, as an initial solution, we first used the intensity threshold technique [20] (Support material 1 in Additional file 1), which is appropriate when dealing with complex objects of unknown topology because it does not rely on a predefined object shape. Although it could be asserted that basic histogram approaches are sensitive to variations specific to MRI, in the segmentation process (stages 1 through 3), we further used a combination of the multiple strategies, including dynamic threshold calculations, contrast analysis, and repairing techniques, to overcome these limitations. In stage 1, the use of the local contrast analysis [21] initialized with the intensity analysis result, enhanced the stability to intraimage IIH. Moreover, in the segmentation process, alternating between 3D and 2D approaches to compute global and local thresholds allows the IIH to be addressed efficiently. To enhance the precision of segmented objects, a final repairing step was added at the end of stages 1 and 3. Finally, using anatomic landmarks in stage 3 allows a strong anatomy-based filtering procedure and also helps to address the previously mentioned problem. In this stage, the intensity threshold was determined by histogram analysis in two recursive steps. Although this technique could lead to an oversegmentation of the fluid, this process was chosen, as it refines the segmentation without discarding any object of interest. Of note, oversegmentation, which does not produce larger objects but selects more objects than those of interest, was addressed by an anatomic filtering to exclude unrelated objects.

We chose segmentation techniques based on basic intensity and contrast analysis because other described methods, including deformable model [22] and level-set [23], do not allow segmentation that adequately reflects the signal nor do they achieve a good stability or precise analysis. These latter approaches can lead to model overfit; they do not implement disease characteristics and are often overconstrained by basic geometric knowledge. Moreover, the parameter values of these methods remain specific to each image quality, thus requiring a manual adjustment to obtain the optimal result. Finally, even if the bone segmentation could be performed with such methods, for the segmentation of the joint effusion, uncertainties including location and topology could not be overcome.

For the joint effusion volume calculation, we used a mesh model approach providing subvoxel precision, in contrast to the conventional approach using voxel size [13,16], which does not take into consideration the effect of sampling (that is, aliasing, introduced by MRI on a real object surface).

Validation experiments using calibrated phantoms for small as well as large volumes gave excellent coefficients of variation. However, although the difference was very small between the phantom volume quantitated with the developed automated system and the reference volume, such a difference could be explained by the following. First, the calibration of the MRI apparatus plays a major role in the volume computed in the image. Even with a small modification of the calibration, the computed volume could slightly differ from the theoretic value. Moreover, this could also be due to the difficulty in classifying partial volume voxels that are on the edge of the fluid object. To reduce the noise in the segmentation process, we used the threshold strategy, which could provide a slightly higher but more stable threshold, causing a slightly less segmented volume. Moreover, in the case of the cylinder phantom, complementary analyses revealed that when the axes of the two cylinders were not perpendicular or parallel to the MRI acquisition direction, the partial volume effect was higher, and therefore, the segmented volume was slightly lower. However, because of its shape, this orientation issue does not exist for the sphere phantom. This could, at least in part, explain the slightly better coefficient of variation of the sphere phantoms than that of the cylinder phantoms when compared with the automated system.

Although the developed automated and manual quantifications used different segmentation processes and a slightly different volume calculation (subvoxel versus voxel size), comparison revealed high statistical correlation, suggesting that the developed automated system is reliable and provides precise joint effusion volume. However, the mean joint effusion volume quantitated by the automated system was slightly lower than the manual computation, probably resulting from an overevaluation of volume from the manual technique. The manual segmentation is a local and 2D process in which the threshold is different for each site in the same image, and in the 2D process, because of the slice thickness, each image is considered as a separate signal; thus a loss of space continuity could occur between images. This could have led to an oversegmentation in which small-object selection was not always consistent between adjacent slices.

In the segmentation field, an automated system is always preferable to a manual one. More specifically, for the joint effusion quantification, it has been reported that manual methods demonstrated intra- and interobserver variations in the range of 11% to 18% [13,16]. Such variations are prevented by an automated method, which also improves the precision of the value and reduces the standard deviation, which is important in clinical trials.

Comparison between the direct aspiration and the automated volume quantification of the MR images yielded a higher volume for the latter, but with an excellent correlation coefficient. Although the direct aspiration method should represent the amount of fluid in the joint, considerable difficulties are associated with complete drainage of the joint cavity [1]. Moreover, it is also well known that after external manipulation of the joint, further joint fluid can sometimes be recovered; this is particularly true when the joint volume is small. This spurious low aspirated volume may result from, among other factors, rice bodies or synovial membrane villi that may obstruct the aspiration [24], or popliteal cysts sequestered by one-way valves [25]. Other factors such as posterior recesses and the complexity of knee joint geometry clearly render portions of the intraarticular space inaccessible. Moreover, our data concur with those obtained with other methods. Wallis *et al. *[5] showed that the OA knee joint effusion volume calculated with a radiolabeled albumin distribution technique was about twice that obtained with direct aspiration. For rheumatoid arthritis patients [5], the volume obtained with manual MRI quantification was more than three times higher than the aspirated joint effusion volume. The latter data also concur with another study in which the calculated residual joint volume in rheumatoid arthritis patients was about 48% [26].

## Conclusions

Joint effusion is a common finding in OA patients and may be related to the activity of the disease. Therefore, noninvasive fully automated quantification of joint effusion volume in the knee would be a valuable tool for diagnostic, follow-up, and clinical studies. The reported automated system for joint effusion volume quantification validated by external means (calibrated phantoms), manual MRI quantification, and direct aspiration was shown to be accurate and precise, in addition to preventing intra- and interobserver variations. The responsiveness to change of such automated quantitative evaluation of joint effusion should be further tested in a longitudinal study in view of its future application in clinical research.

## Abbreviations

IIH: intensity inhomogeneity; MRI: magnetic resonance imaging; OA: osteoarthritis.

## Competing interests

JP Pelletier and J Martel-Pelletier are consultants for and shareholders in ArthroVision Inc. JP Raynauld, M Dorais, and MA d'Anjou are consultants for ArthroVision Inc. W Li and F Abram are employees of ArthroVision Inc.

## Authors' contributions

All authors read and approved the contents of this final version of the manuscript. WL, FA, JPP, JPR, MAD, and JMP contributed to study design. WL, FA, and JPR contributed to acquisition of data. WL, FA, JPP, and JMP contributed to analysis and interpretation of data. WL, FA, JPP, JPR, MD, MAD, and JMP contributed to manuscript preparation. JPR and MD contributed to statistical analysis.

## Supplementary Material

Additional file 1 **Support material**. Description of intensity histogram based thresholding technique, local contrast technique, and repairing process. Click here for file
