# Supplementary material for: Fully automated system for the quantification of human osteoarthritic knee joint effusion volume using magnetic resonance imaging
Source: Arthritis Res Ther. 2010 Sep 16;12(5):R173. doi: 10.1186/ar3133 (PMC2991000; doi:10.1186/ar3133)
Supplement: Additional file 1 — Support material. Description of intensity histogram based thresholding technique, local contrast technique, and repairing process. [file ar3133-S1.PDF]

SUPPORT MATERIAL

1. Intensity histogram based thresholding technique

Intensity histograms to determine threshold value are often used in image processing[1]. An intensity histogram is a graphic that displays the number of pixels/voxels for each intensity interval in 2D/3D images. It gives information about the distribution of intensity levels of the image. The thresholding, a common image segmentation method, refers to finding a threshold to divide an image into two classes, object and background.

To automatically determine a threshold value based on a histogram, one technique is to search a critical point on the histogram curve to provide an optimal threshold value[1]. This optimal point usually corresponds to an inflection point P_T (Figure A) whose corresponding intensity value is also the optimal threshold T for a specific application. In brief, the detection is achieved by identifying the furthest point P_T of the histogram from a reference line, that is, the point having the maximum distance D_{MAX}. The reference line L_{REF} links two characteristic points (P_{MIN} and P_{MAX}) of the histogram that defines the intensity interval of analysis [T_{MIN}, T_{MAX}]. One characteristic point is defined by the start or end point of the histogram, and the other one is the peak or half peak point. The application of this technique can be used once or recursively to refine the search. All these options are selected based on the intensity range of the object of interest and are defined during the system development.

1. Nacereddine N, Hamami MT, Oucief N. **Non-parametric histogram-based thresholding methods for weld defect detection in radiography.** *Proceedings of World Academy of Science, Engineering and Technology* 2005;**9:**213-7.

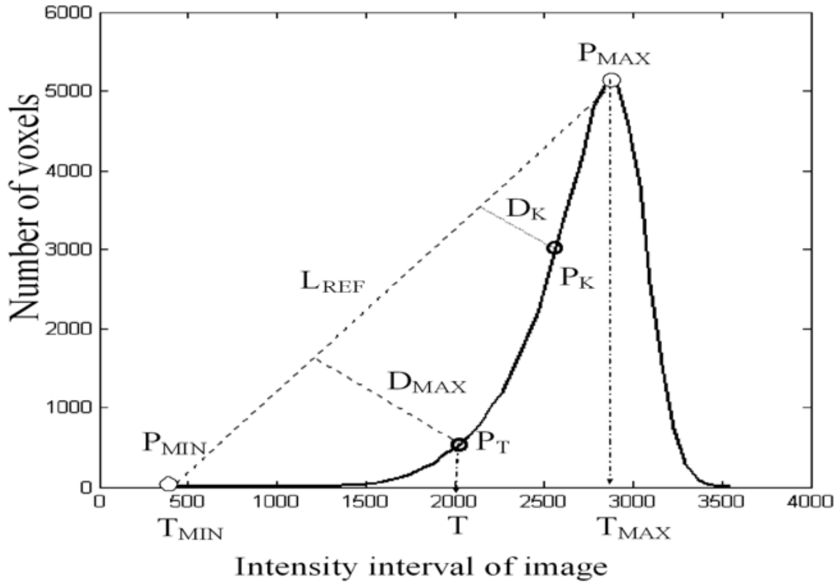

Figure A. Illustration of the automatic determination of a threshold value *T* based on the intensity histogram, which is achieved by detecting point P_T that has the maximum distance D_{MAX} among D_K corresponding to the distances between all points $P_K \in [P_{MIN}, P_{MAX}]$ on the curve segment and the reference line L_{REF} defined between [T_{MIN}, T_{MAX}].

2. Local contrast technique

The 2D local contrast analysis serves to locate more precisely the interface between bone and surrounding tissues. For optimal efficacy of the processing, the computation of the local contrast must be performed along the bone wall. The images are thus resampled in a cylindrical coordinate system (radius ρ, angle θ, height h) using the gravity center of the points of the initial solution from the thresholding as the origin in each image. The local contrast evaluation uses a 5x5 pixel window in the (ρ, h) plane. Note that in the resampled images, the inside of the bone is easily defined, enabling computation of the local contrast characteristics for each pixel of the bone, i.e. the mean M and the standard deviation S, permitting the evaluation of a threshold, $T = M + 3\times S$, used to filter the images.

3. Repairing process

This is conducted from the distal to the proximal for the femur and inversely for the tibia, in order to process the best contrasted (centre) images first. This allows the propagation of reliable information between slices. The criterion used to identify a partial or missing object is the object area ratio between the current and the previous slices. Thus, a ratio less than 50% determines the necessity to run a repairing procedure. In this situation, local intensity properties, mean M and standard deviation S, from pixels located inside the mask copied from the previous slice are calculated. The local low T_L and high T_H thresholds are re-evaluated based on $M \pm 2 \times S$ and a 2D filtering is performed.
